# Technical Refinements in Laparoscopic Transabdominal Preperitoneal Repair for Scrotal Hernia Using High Sac Transection and Internal Ring Closure

**DOI:** 10.7759/cureus.102590

**Published:** 2026-01-29

**Authors:** Guangbin Chen, Yanguang Sha, Guangming Xu, Ke Wang, Yuzhi Hu

**Affiliations:** 1 Department of Hepato-Pancreato-Biliary Surgery, The Second People's Hospital of Wuhu, Wuhu Hospital Affiliated to East China Normal University, Wuhu, CHN; 2 Graduate School, Wannan Medical College, Wuhu, CHN

**Keywords:** high sac transection, inguinoscrotal hernia, internal ring closure, laparoscopic hernia repair, tapp, transabdominal preperitoneal repair

## Abstract

Inguinoscrotal hernia is a challenging subtype of inguinal hernia due to its large hernia sac, scrotal extension, and increased risk of postoperative complications, including seroma formation and recurrence. Although laparoscopic transabdominal preperitoneal (TAPP) repair is widely accepted for inguinal hernia management, optimal strategies for hernia sac handling and internal ring reconstruction in scrotal hernias remain controversial. We report the case of a 54-year-old man with a right inguinoscrotal hernia who underwent laparoscopic TAPP repair incorporating high transection of the hernia sac combined with internal ring closure. The procedure was completed successfully without intraoperative complications. The postoperative course was uneventful, and no recurrence, chronic pain, or scrotal seroma was observed during nine months of follow-up. This case suggests that high sac transection combined with anatomical narrowing of the internal ring may simplify the surgical procedure, reduce tissue trauma, and enhance repair stability in selected patients with inguinoscrotal hernia.

## Introduction

Inguinal hernia is one of the most common surgical conditions worldwide [1]. Inguinoscrotal hernia, characterized by the descent of the hernia sac into the scrotum, represents a more complex variant associated with larger defects, extensive dissection, and a higher incidence of postoperative complications [2]. Patients often experience progressive scrotal enlargement, discomfort, and functional impairment, which significantly affect quality of life.

With advances in minimally invasive surgery, laparoscopic techniques such as transabdominal preperitoneal (TAPP) and totally extraperitoneal (TEP) repair have become standard options for inguinal hernia repair [3]. Compared with open surgery, laparoscopic approaches provide superior visualization of the myopectineal orifice, reduced postoperative pain, and faster recovery [4,5]. However, laparoscopic repair of inguinoscrotal hernias remains technically demanding. Traditional management of large hernia sacs often requires extensive dissection along the spermatic cord, which may increase operative time, bleeding, and the risk of seroma, ischemic orchitis, and chronic pain [6].

Recent trends in hernia surgery emphasize functional reconstruction and tissue preservation rather than complete excision of the hernia sac [7]. In this report, we describe a refined TAPP technique combining high hernia sac transection with internal ring closure, aiming to reduce operative complexity, minimize tissue trauma, and improve postoperative outcomes. We present a representative case and discuss the rationale, technical considerations, and potential benefits of this approach.

## Case presentation

A 54-year-old man presented with a progressively enlarging mass in the right inguinal region that had been present for more than one year. The mass was initially asymptomatic and fully reducible, disappearing in the supine position. Over time, the frequency of protrusion increased, prompting the patient to seek medical evaluation.

The patient had a history of type 2 diabetes mellitus managed with metformin and acarbose. He had no prior abdominal surgeries and no significant family history. On physical examination, vital signs were stable. A soft, non-tender mass measuring approximately 10 × 8 cm was noted in the right inguinal region. The mass was completely reducible, with no signs of incarceration or strangulation. A positive cough impulse was elicited upon examination of the right inguinoscrotal region, consistent with the diagnosis of a hernia.

**Figure 1:**
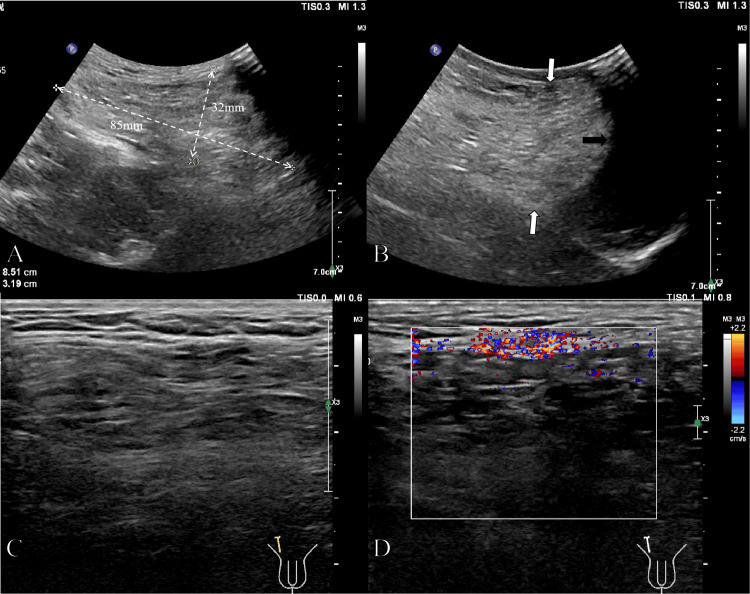
Color Doppler ultrasonography findings. A low-frequency probe was used to evaluate the overall morphology of the hernia sac (A); the inferior margin of the hernia sac (white arrow, B) extended into the scrotum (black arrow, B). High-frequency ultrasonography demonstrated linear hyperechoic structures within the hernia sac consistent with omental tissue (C), and Color Doppler imaging revealed a few intralesional blood flow signals (D).

Color Doppler ultrasonography revealed a heterogeneous mass in the right inguinal region measuring approximately 85 × 32 mm during deep inspiration. The proximal portion communicated with the abdominal cavity, while the distal portion extended into the scrotum. Omental tissue was identified within the hernia sac (Figure 1).

**Figure 2:**
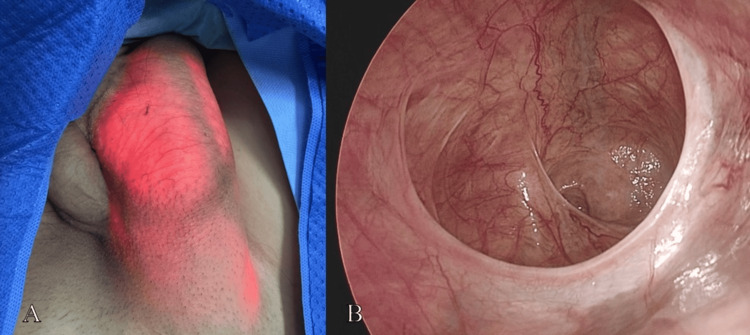
Intraoperative exploration of the hernia sac. The endoscope was advanced into the hernia sac. Externally, the hernia sac appeared as a "red lantern-like" structure (A). Intra-abdominal visualization demonstrated extension of the hernia sac into the scrotum (B).

Based on the clinical and imaging findings, a diagnosis of right inguinoscrotal hernia was established. After obtaining informed consent, the patient underwent laparoscopic TAPP repair under general anesthesia. Three trocars were placed using a standard technique. Intraoperative exploration revealed an indirect hernia defect measuring approximately 3.0 × 2.5 cm medial to the right inferior epigastric vessels, with the hernia sac extending into the scrotum (Figure 2).

**Figure 3:**
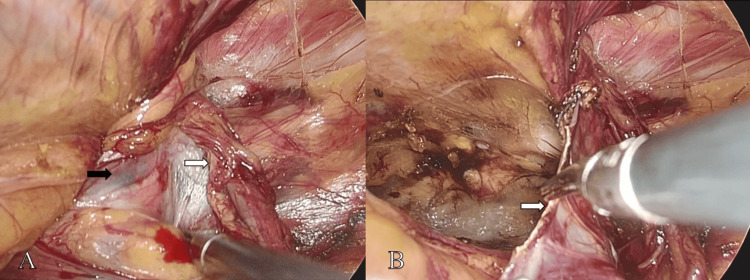
High transection of the hernia sac. At the level of the sac neck, the hernia sac (black arrow, A) was dissected free from the spermatic cord (white arrow, A). High transection of the hernia sac was then performed (white arrow, B).

No contralateral hernia was identified. A curvilinear peritoneal incision was made approximately 2 cm above the internal ring. The preperitoneal space was developed, including the spaces of Retzius and Bogros. High transection of the hernia sac was performed at the level of the sac neck (Figure 3), and the distal sac was intentionally left in situ to avoid extensive dissection along the spermatic cord. The preperitoneal dissection was extended sufficiently to expose the entire myopectineal orifice.

**Figure 4:**
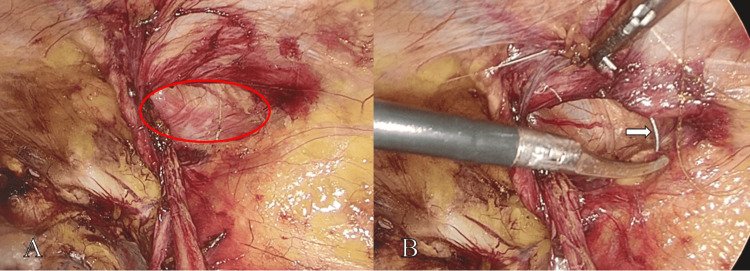
Internal ring closure technique. The internal ring defect measured approximately 3.0 × 2.5 cm (red ellipse, A). A barbed suture was used to narrow the internal ring (white arrow, B).

The enlarged internal ring was then narrowed to approximately 1.0 cm using a continuous barbed suture (Figure 4), with careful preservation of the spermatic cord structures. A three-dimensional polypropylene mesh (approximately 10 × 8 cm) was placed to cover the myopectineal orifice, and the peritoneum was closed with a continuous absorbable suture. The operative time was 50 minutes, with an estimated blood loss of 10 mL.

The postoperative course was uneventful. The patient resumed oral intake on postoperative day 1 and was discharged on postoperative day 4 without complications. At nine-month follow-up, he remained asymptomatic, with no evidence of recurrence, chronic pain, or scrotal seroma.

## Discussion

Laparoscopic TAPP repair is a well-established and effective approach for inguinal hernia repair. However, inguinoscrotal hernias pose distinct technical challenges, primarily related to management of the large hernia sac and reconstruction of the enlarged internal ring [8]. Conventional laparoscopic strategies often involve complete dissection and reduction of the hernia sac, which may lead to prolonged operative time, increased bleeding, and a higher incidence of postoperative complications [9].

High hernia sac transection

High transection of the hernia sac at the level of the sac neck represents a tissue-sparing strategy that prioritizes functional repair over extensive dissection [10]. By eliminating communication between the peritoneal cavity and the distal sac, this technique effectively addresses the hernia defect while avoiding unnecessary manipulation of the spermatic cord [11]. Preservation of the distal sac reduces surgical trauma and may lower the risk of postoperative seroma, ischemic orchitis, and chronic pain, complications that are particularly relevant in large scrotal hernias [9].

In the present case, the hernia sac extended deeply into the scrotum, making complete distal dissection both unnecessary and potentially harmful. High sac transection allowed safe and efficient management of the hernia sac while minimizing operative complexity.

Internal ring closure

An enlarged internal ring is a defining feature of indirect inguinal and inguinoscrotal hernias. Mesh placement alone may not fully compensate for a markedly widened internal ring, potentially predisposing to mesh migration or recurrence [12]. Anatomical narrowing of the internal ring restores the physiological configuration of the inguinal canal and provides a stable foundation for mesh reinforcement [13].

In this case, controlled suturing reduced the internal ring to near-physiological dimensions while preserving spermatic cord perfusion. This step transformed the repair from passive defect coverage into active anatomical reconstruction, which may enhance long-term durability.

Case-specific challenges

Several challenges were present in this patient. The large scrotal extension of the hernia increased the risk of postoperative seroma, while the patient’s history of diabetes mellitus raised concerns regarding wound healing. Additionally, internal ring closure required meticulous tension control to avoid compromising the spermatic cord. These factors underscore the importance of individualized surgical planning and careful intraoperative technique.

Limitations

As a single case report, this study cannot establish definitive conclusions regarding long-term outcomes or general applicability. Further prospective studies with larger patient cohorts and longer follow-up are needed to validate the safety and efficacy of this combined approach.

## Conclusions

In conclusion, this report demonstrates the feasibility of high hernia sac transection combined with internal ring closure during laparoscopic TAPP repair for a selected patient with an inguinoscrotal hernia. This technical refinement simplified sac management and appeared to facilitate anatomical reconstruction in this single case. However, definitive conclusions regarding its efficacy in reducing postoperative complications and recurrence require prospective comparative studies with longer follow-up.
